# Supplementary material for: A hybrid model connecting regulatory interactions with stem cell divisions in the root
Source: Quant Plant Biol. 2021 Apr 12;2:e2. doi: 10.1017/qpb.2021.1 (PMC10095808; doi:10.1017/qpb.2021.1)
Supplement: Supplementary file 1 [file S2632882821000011sup001.zip › S2632882821000011supp001.pdf]

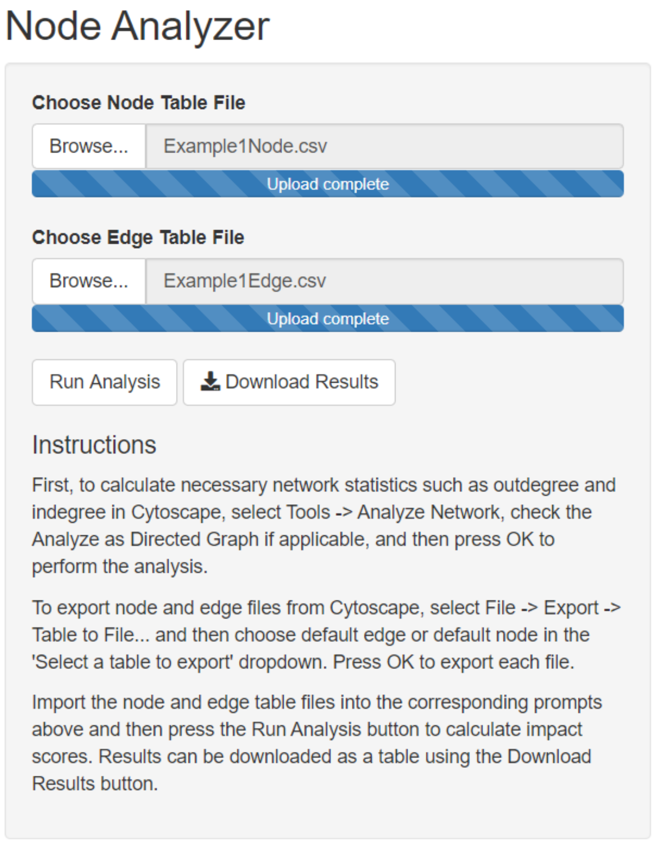

Supplemental Figure 1 – Screenshot of the Node Analyzer user interface. Node Analyzer is available at https://rspurney.shinyapps.io/nodeanalyzer/.
