# Supplementary material for: A hybrid model connecting regulatory interactions with stem cell divisions in the root
Source: Quant Plant Biol. 2021 Apr 12;2:e2. doi: 10.1017/qpb.2021.1 (PMC10095808; doi:10.1017/qpb.2021.1)
Supplement: Supplementary file 1 [file S2632882821000011sup001.zip › S2632882821000011supp002.pdf]

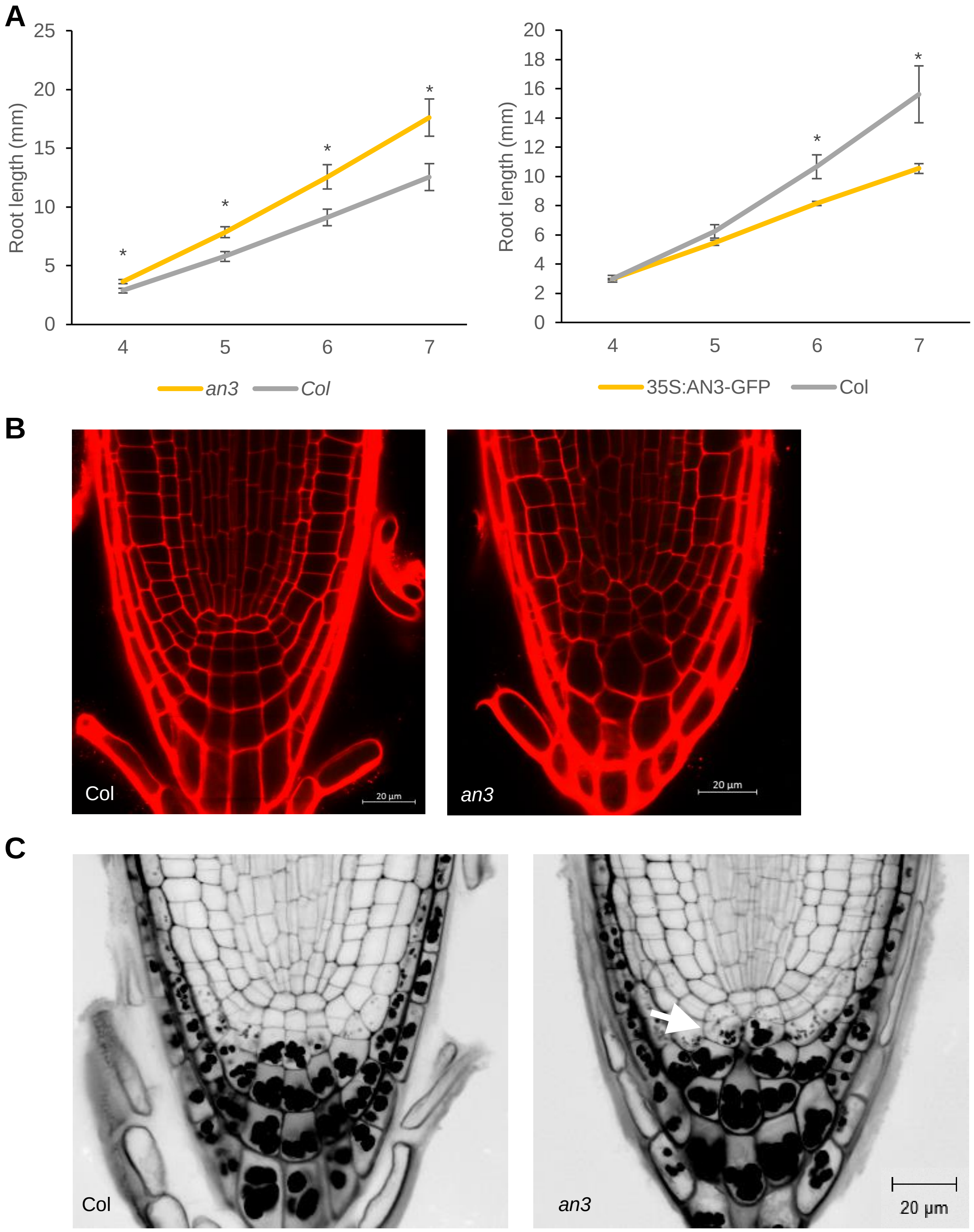

Supplemental Figure 2 – Phenotypical analysis of AN3 loss- and gain-of-function lines. (A) Root length of 4- to 7-day-old *an3* (left) and 35S:AN3-GFP (right) lines. (B) Confocal microscope images of WT (Col) and *an3* mutants. Scale bar = 20 µm. (D) Confocal microscope images of mPSPI stained roots and images of WT (Col) and *an3* mutants. White arrow indicates starch granules. Data are presented as mean ± SEM. * = $p<0.05$ (Student's t-test).
