# Supplementary material for: A hybrid model connecting regulatory interactions with stem cell divisions in the root
Source: Quant Plant Biol. 2021 Apr 12;2:e2. doi: 10.1017/qpb.2021.1 (PMC10095808; doi:10.1017/qpb.2021.1)
Supplement: Supplementary file 1 [file S2632882821000011sup001.zip › S2632882821000011supp003.pdf]

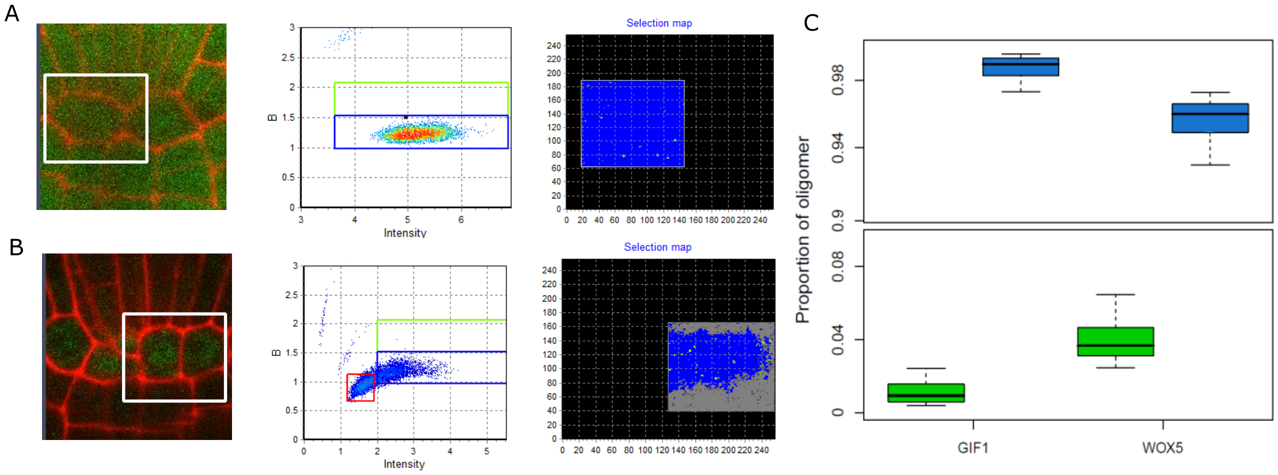

Supplemental Figure 3 – Oligomeric state of WOX5 and AN3 in the QC. Number and brightness analysis on AN3 (A) and WOX5 (B) translational fusions. (A,B) Representative image of the root stem cell niche of *an3*xpAN3:AN3-GFP and *wox5*xpWOX5:WOX5-GFP (left), brightness vs intensity plot (middle), and false-colored map (right). The white box represents a 128x128 pixel region of interest used for analysis. In the false-colored image, grey represents background, blue represents monomer, and green represents dimer. (C) Percentage of monomer (blue) and dimer (green) in *an3*xpAN3:AN3-GFP and *wox5*xpWOX5:WOX5-GFP.
