# Supplementary material for: A hybrid model connecting regulatory interactions with stem cell divisions in the root
Source: Quant Plant Biol. 2021 Apr 12;2:e2. doi: 10.1017/qpb.2021.1 (PMC10095808; doi:10.1017/qpb.2021.1)
Supplement: Supplementary file 1 [file S2632882821000011sup001.zip › S2632882821000011supp004.pdf]

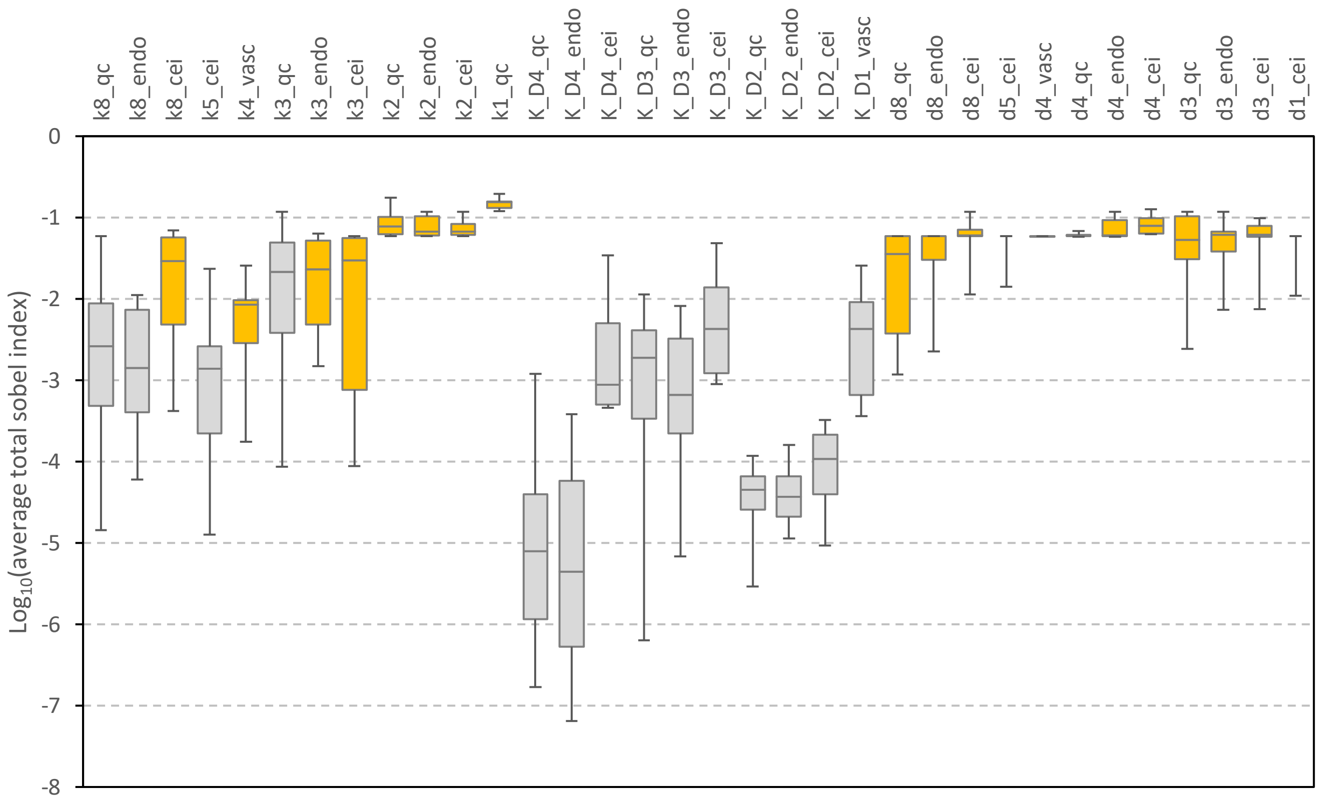

Supplemental Figure 4 – Sensitivity analysis of the hybrid stem cell division model. Box plots for the 34 parameters of the Total Sobol Index of 10 technical replicates. Yellow box plots are parameters that have a significant sensitivity compared to K_D2_qc ($p < 0.05$, Student's t-test). Data are presented as mean ± SEM.
