# Supplementary material for: A hybrid model connecting regulatory interactions with stem cell divisions in the root
Source: Quant Plant Biol. 2021 Apr 12;2:e2. doi: 10.1017/qpb.2021.1 (PMC10095808; doi:10.1017/qpb.2021.1)
Supplement: Supplementary file 1 [file S2632882821000011sup001.zip › S2632882821000011supp005.pdf]

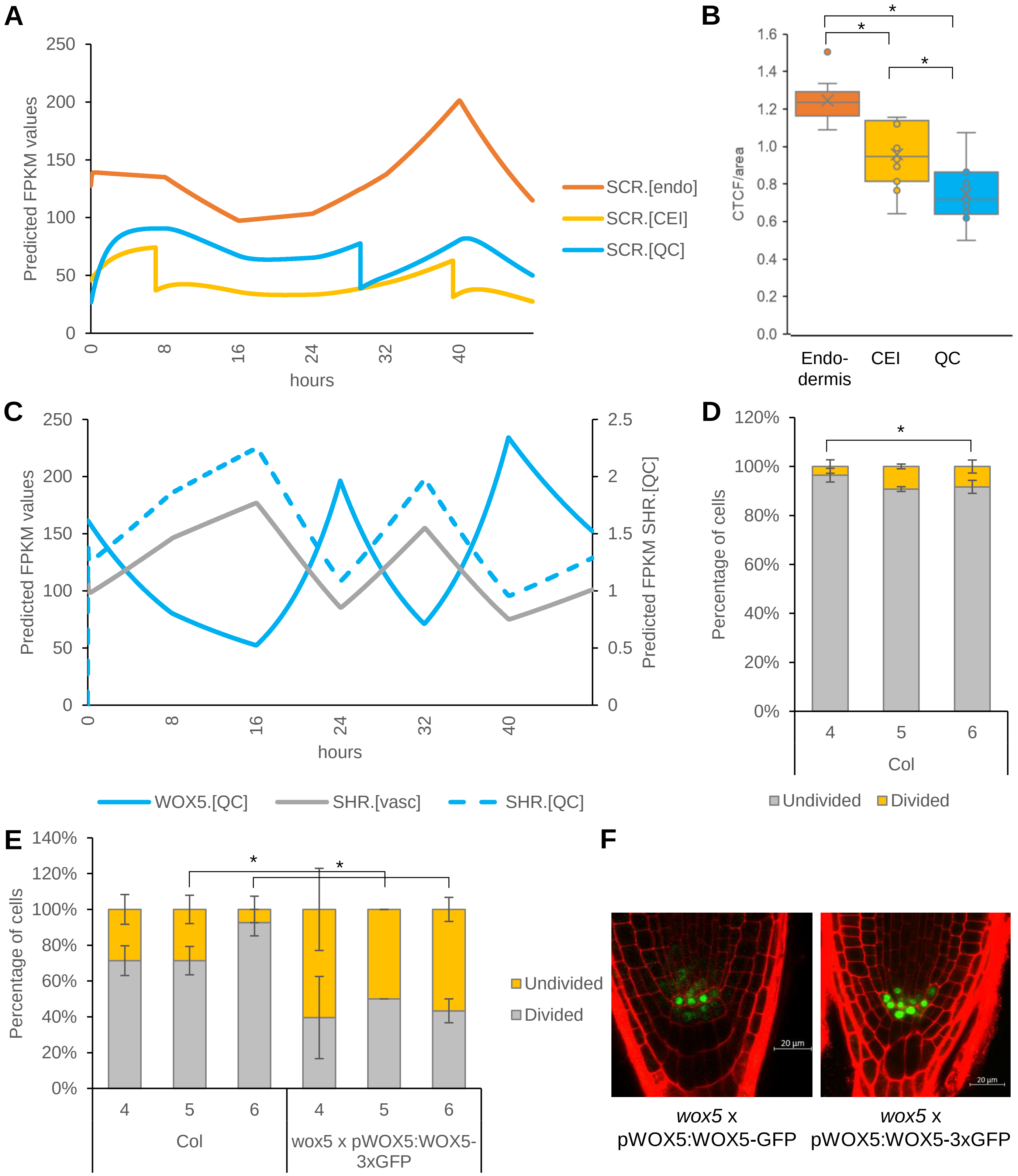

Supplemental Figure 5 – Model predictions and experimental validation. (A) Model predictions of *SCR* expression in endodermis, CEI and QC. (B) CTCF of SCR at 5 days and 16 hours (40 hour time point in the model predictions) in the endodermis, CEI, and QC. (C) Model simulations of *WOX5* and *SHR* expression in the vascular initials and QC without implementing QC divisions in the hybrid model. (D) Percentage of divided QC 4-, 5-, and 6-day-old wild-type roots. (E) Percentage of divided and undivided CEI cells in 4-, 5-, and 6-day-old wild-type (Col) and *wox5*xpWOX5:WOX5-3xGFP roots. (F) Representative images of the root tip of (left) *wox5*xpWOX5:WOX5-3xGFP and (right) *wox5*xpWOX5:WOX5-3xGFP. * = $p < 0.05$ (D: Student's t-test with 4 days as control, B,E: Wilcoxon with Steel Dwass). QC = quiescent center, CEI = cortex endodermis initial, SCR = SCARECROW, SHR = SHORTROOT, WOX5 = WUSCHEL-RELATED HOMEOBOX 5.
