# Supplementary material for: A hybrid model connecting regulatory interactions with stem cell divisions in the root
Source: Quant Plant Biol. 2021 Apr 12;2:e2. doi: 10.1017/qpb.2021.1 (PMC10095808; doi:10.1017/qpb.2021.1)
Supplement: Supplementary file 1 [file S2632882821000011sup001.zip › S2632882821000011supp006.pdf]

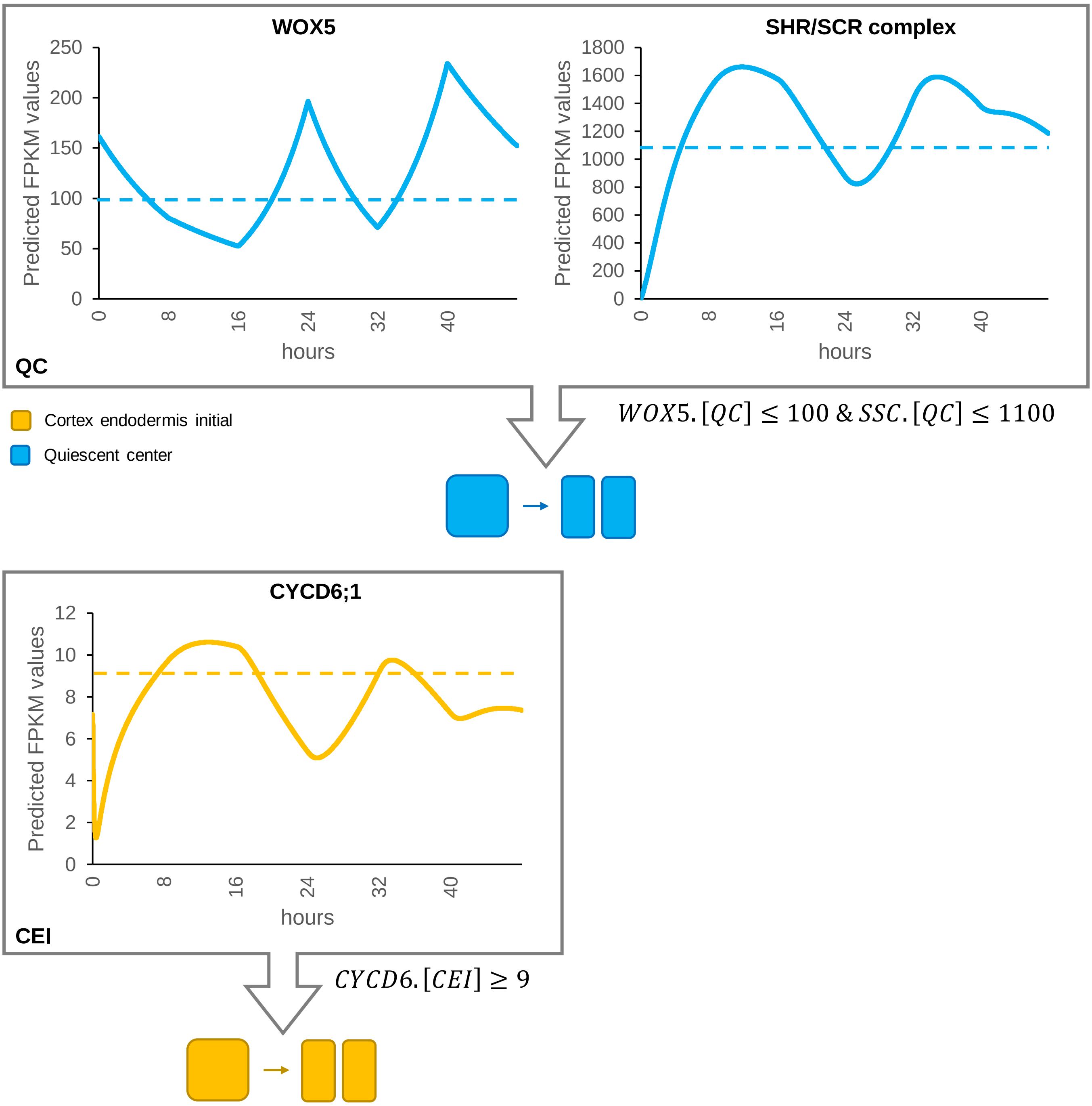

Supplemental Figure 6 – Mathematical modeling of WOX5, SHR/SCR, and CYCD6;1. Model simulation of the estimated expression of *WOX5*, *SHR/SCR*, and *CYCD6;1* in the QC and CEI. Low levels of the *SHR/SCR* complex and low levels of *WOX5* are considered to trigger QC divisions. High levels of *CYCD6;1* are considered to trigger CEI divisions.
