# Supplementary material for: A hybrid model connecting regulatory interactions with stem cell divisions in the root
Source: Quant Plant Biol. 2021 Apr 12;2:e2. doi: 10.1017/qpb.2021.1 (PMC10095808; doi:10.1017/qpb.2021.1)
Supplement: Supplementary file 1 [file S2632882821000011sup001.zip › S2632882821000011supp007.pdf]

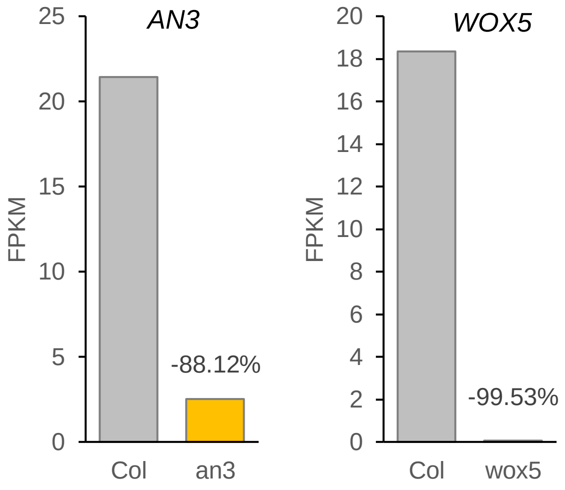


**Supplemental Figure 7 – Gene expression in *an3* and *wox5* loss-of-function lines.** Expression levels of *AN3* and *WOX5* in *an3* and *wox5* loss-of-function lines, respectively. WOX5 = WUSCHEL-RELATED HOMEOBOX 5, AN3 = ANGUSTIFOLIA 3.
