# Supplementary material for: A hybrid model connecting regulatory interactions with stem cell divisions in the root
Source: Quant Plant Biol. 2021 Apr 12;2:e2. doi: 10.1017/qpb.2021.1 (PMC10095808; doi:10.1017/qpb.2021.1)
Supplement: Supplementary file 1 [file S2632882821000011sup001.zip › S2632882821000011supp008.pdf]

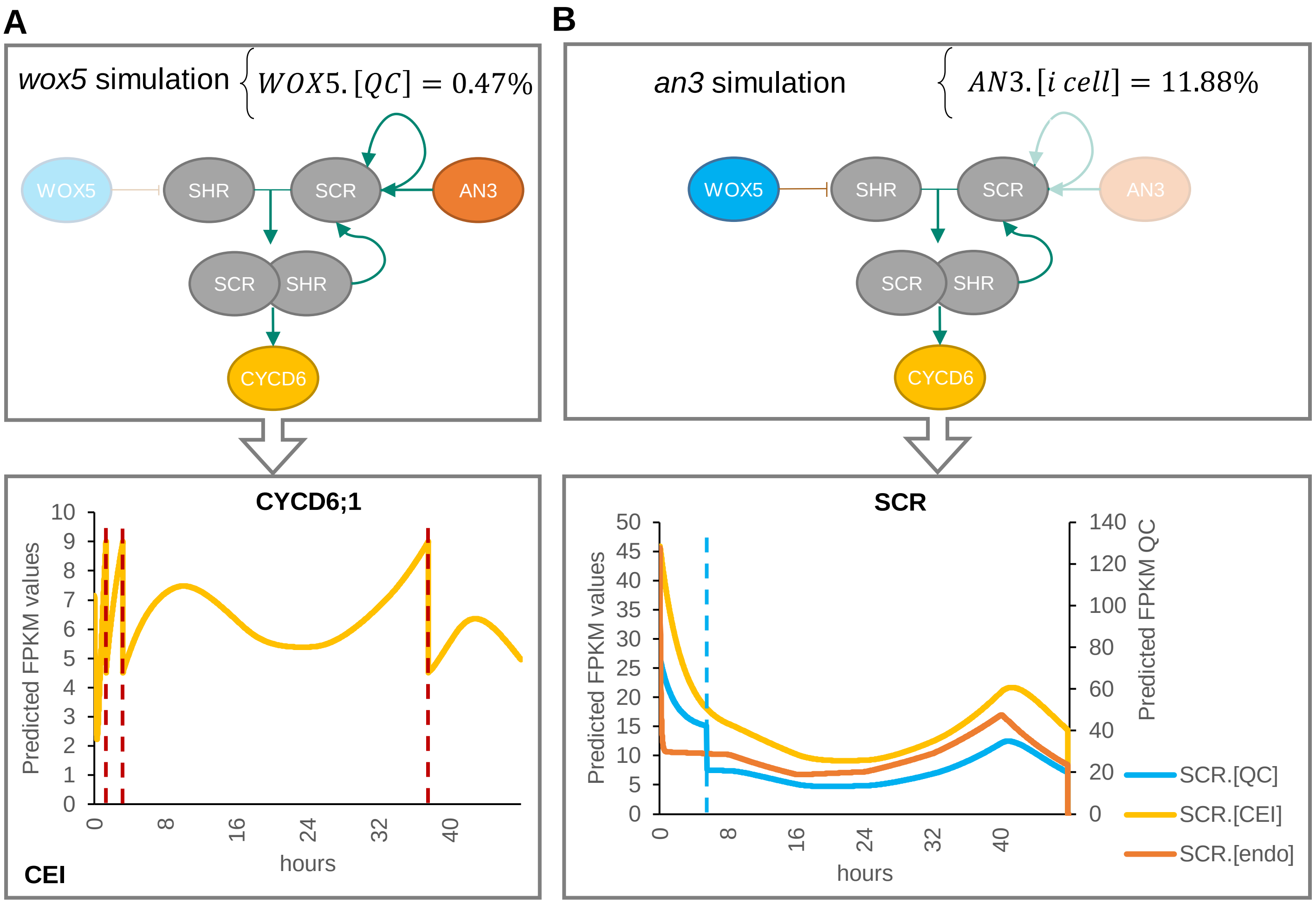

Supplemental Figure 8 – Mathematical modeling of *wox5* and *an3* loss-of-function. Top panels indicate the model setting to simulate *wox5* (A) or *an3* (B) loss-of-function. Bottom panel shows a model simulation of the estimated expression of *CYCD6;1* and *SCR* in the QC, CEI, or endodermal cell (endo) in a *wox5* (A) or *an3* (B) mutant background. Red dotted lines indicate CEI divisions and the blue dotted line indicates the time point of the QC division.
