# Supplementary material for: A hybrid model connecting regulatory interactions with stem cell divisions in the root
Source: Quant Plant Biol. 2021 Apr 12;2:e2. doi: 10.1017/qpb.2021.1 (PMC10095808; doi:10.1017/qpb.2021.1)
Supplement: Supplementary file 1 [file S2632882821000011sup001.zip › S2632882821000011supp009.pdf]

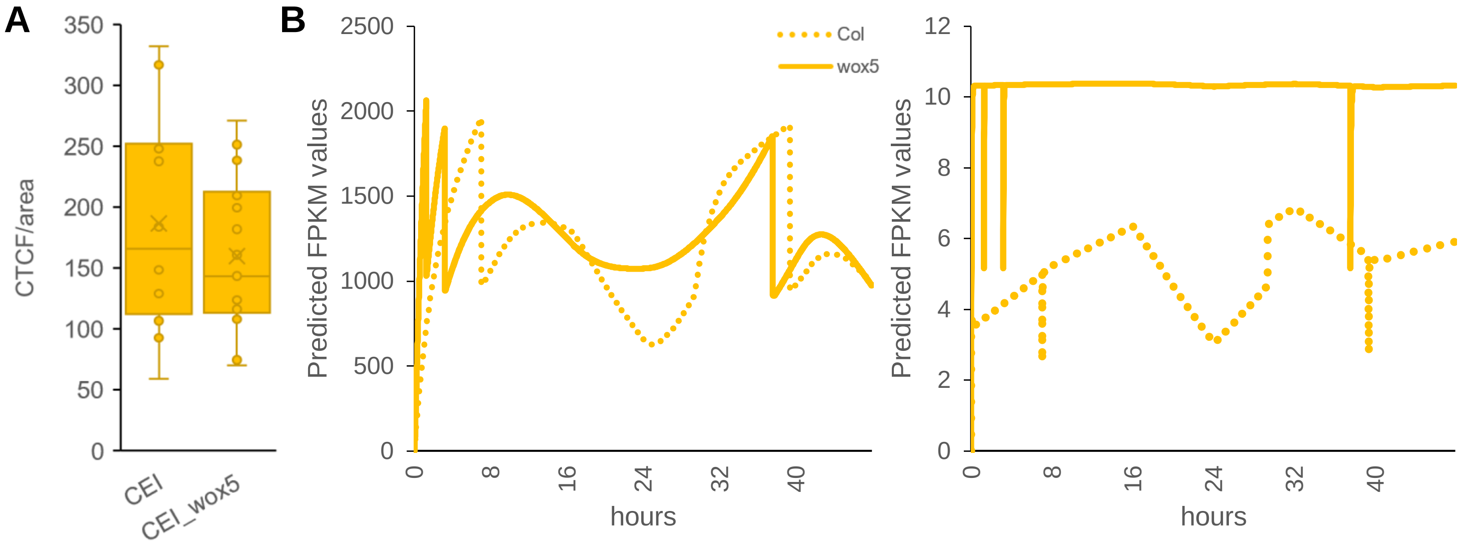

Supplemental Figure 9 – Protein quantification in *wox5* loss-of-function line. (A) Corrected total cell fluorescence (CTCF) measurements of SHR at 5 days (24 hour time point in the model predictions) in the CEI of pSHR::SHR-GFP (left) or pSHR::SHR-GFP x wox5 (right). * = $p < 0.05$ (Wilcoxon test). (B) Model simulations of the estimated expression of SHR/SCR complex (left) and SHR (right) in the CEI in a wild-type or *wox5* mutant background. CEI = cortex endodermis initial, SHR = SHORTROOT, SCR = SCARECROW, WOX5 = WUSCHEL-RELATED HOMEOBOX 5.
