# Supplementary material for: A hybrid model connecting regulatory interactions with stem cell divisions in the root
Source: Quant Plant Biol. 2021 Apr 12;2:e2. doi: 10.1017/qpb.2021.1 (PMC10095808; doi:10.1017/qpb.2021.1)
Supplement: Supplementary file 1 [file S2632882821000011sup001.zip › S2632882821000011supp010.pdf]

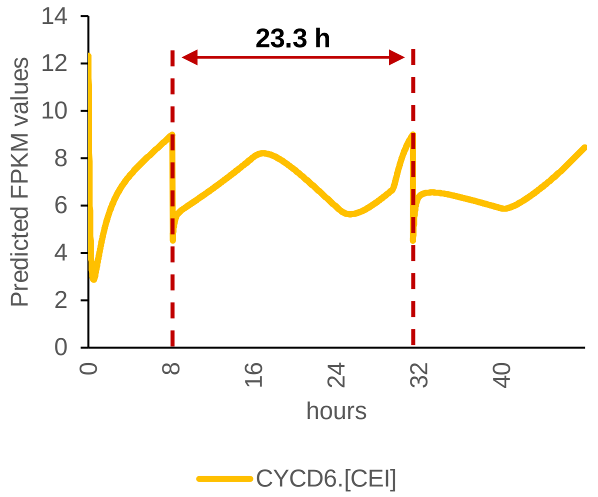


**Supplemental Figure 10 – Mathematical modeling of *CYCD6;1* expression.** Model simulation of the estimated expression of *CYCD6;1* in the CEI in a wild-type background. Red dotted lines indicate a division in the CEI.
