# Supplementary material for: A hybrid model connecting regulatory interactions with stem cell divisions in the root
Source: Quant Plant Biol. 2021 Apr 12;2:e2. doi: 10.1017/qpb.2021.1 (PMC10095808; doi:10.1017/qpb.2021.1)
Supplement: Supplementary file 1 [file S2632882821000011sup001.zip › S2632882821000011supp017.docx]

| Parameter | Biological meaning | Value | | | |
| --- | --- | --- | --- | --- | --- |
|  |  | QC | CEI | Endodermis | Vascular initial |
| k8 | SHR-SCR production rate | 5.00  (Clark et al., 2020) | 5.00  (Clark et al., 2020) | 5.00  (Set to fix value) | Not produced |
| k5 | CYCD6;1 production rate | Not produced | 25000.00  (Set to fix value) | Not produced | Not produced |
| k4 | SHR production rate | Not produced | Not produced | Not produced | 6850.56995  (Estimated) |
| k3 | SCR production rate | 268.57  (Clark et al., 2020) | 77.86  (Clark et al., 2020) | 20186.461  (Estimated) | Not produced |
| k2 | AN3 production rate | Time dependent | Time dependent | Time dependent | Not modeled |
| k1 | WOX5 production rate | Time dependent | Not produced | Not produced | Not produced |
| K_D4 | Dissociation constant for SHR | 1000.00  (Clark et al., 2020) | 1000.00  (Clark et al., 2020) | 1000.00  (Set to fix value) | Not modeled |
| K_D3 | Dissociation constant for SCR | 600.00  (Clark et al., 2020) | 600.00  (Clark et al., 2020) | 600.00  (Set to fix value) | Absent |
| K_D2 | Dissociation constant for AN3 | 500.00  (Set to fix value) | 500.00  (Set to fix value) | 1000.00  (Set to fix value) | Absent |
| K_D1 | Dissociation constant for WOX5 | Not modeled | Not modeled | Not modeled | 3.00  (Clark et al., 2020) |
| d8 | SHR-SCR degradation rate | 0.50  (Clark et al., 2020) | 0.883631329  (Estimated) | 45.4204051  (Estimated) | Not produced |
| d5 | CYCD6;1 degradation rate | Not produced | 7.962335  (Estimated) | Not produced | Not produced |
| d4 | SHR degradation rate | 259.9675969  (Estimated) | 130.8429372  (Estimated) | 109.0219895  (Estimated) | 12.26685298  (Estimated) |
| d3 | SCR degradation rate | 1.00  (Clark et al., 2020) | 0.50  (Clark et al., 2020) | 38.94783205  (Estimated) | Not produced |
| d1 | WOX5 degradation rate | Time dependent | Not modeled | Absent | 397.0117125  (Estimated) |
| A | Cell area | 45.27 µm^2^ | 61.29 µm^2^ | 53.35 µm^2^ | 31.36 µm^2^ |

D_SHR_ (vasc to QC) = 1.73 µm^2^/sec, D_SHR_ (vasc to CEI/endo) = 2.45 µm^2^/sec, D_WOX5_ (QC to vasc) = 11.0418 µm^2^/sec

a_SHR_ (vasc to QC) = 3.3099 /min, a_SHR_ (vasc to CEI/endo) = 4,6875 /min, a_WOX5_ (QC to vasc) = 14,6346 /min

Re-estimation of four former and two new parameters for the second model:

| Parameter | Biological meaning | Value | | | |
| --- | --- | --- | --- | --- | --- |
|  |  | QC | CEI | Endodermis | Vascular initial |
| k3 | SCR production rate | 268.57  (Clark et al., 2020) | 318.56  (Estimated) | 8449.07  (Estimated) | Not produced |
| k6 | Repressor X production rate | Not modeled | 2803.75  (Estimated) | Not modeled | Not modeled |
| K_D6 | Dissociation constant for repressor X | Not modeled | 0.002  (Set to fix value) | Not modeled | Not modeled |
| d3 | SCR degradation rate | 1.00  (Clark et al., 2020) | 0.50  (Clark et al., 2020) | 11.44  (Estimated) | Not produced |
| d5 | CYCD6;1 degradation rate | Not produced | 5.85  (Estimated) | Not produced | Not produced |
| d6 | Repressor X degradation rate | Not modeled | 80.08  (Estimated) | Not modeled | Not modeled |
